# Sex-specific effects of subchronic NMDA receptor antagonist MK-801 treatment on hippocampal gamma oscillations

**DOI:** 10.3389/fnins.2024.1425323

**Published:** 2024-08-07

**Authors:** Tim Simon Neuhäusel, Zoltan Gerevich

**Affiliations:** Institute of Neurophysiology, Charité - Universitätsmedizin Berlin, Corporate Member of Freie Universität Berlin and Humboldt Universität zu Berlin, Berlin, Germany

**Keywords:** electroencephalography (EEG), gamma oscillations, sex-specific, cognition, electrophysiology, ketamine

## Abstract

N-methyl-D-aspartate (NMDA) receptor antagonists are widely used to pharmacologically model schizophrenia and have been recently established in the treatment of treatment-resistant major depression demonstrating that the pharmacology of this substance class is complex. Cortical gamma oscillations, a rhythmic neuronal activity associated with cognitive processes, are increased in schizophrenia and deteriorated in depressive disorders and are increasingly used as biomarker in these neuropsychiatric diseases. The opposite use of NMDA receptor antagonists in schizophrenia and depression raises the question how their effects are in accordance with the observed disease pathophysiology and if these effects show a consequent sex-specificity. In this study in rats, we investigated the effects of subchronic (14 days) intraperitoneal injections of the NMDA receptor antagonist MK-801 at a subanesthetic daily dose of 0.2 mg/kg on the behavioral phenotype of adult female and male rats and on pharmacologically induced gamma oscillations measured *ex vivo* from the hippocampus. We found that MK-801 treatment leads to impaired recognition memory in the novel object recognition test, increased stereotypic behavior and reduced grooming, predominantly in female rats. MK-801 also increased the peak power of hippocampal gamma oscillations induced by kainate or acetylcholine only in female rats, without affecting the peak frequency of the oscillations. The findings indicate that blockade of NMDA receptors enhances gamma oscillations predominantly in female rats and this effect is associated with behavioral changes in females. The results are in accordance with clinical electrophysiological findings and highlight the importance of hippocampal gamma oscillations as a biomarker in schizophrenia and depression.

## 1 Introduction

N-methyl-D-aspartate (NMDA) receptor antagonists such as ketamine, phencyclidine (PCP) and MK-801 are widely used to pharmacologically model schizophrenia (SZ) in animals (Lee and Zhou, 2019). Beyond that, ketamine and MK-801 show antidepressant effects (Trullas and Skolnick, 1990; Adell, 2020; Johnston et al., 2024) demonstrating that the pharmacology of this substance class is complex.

SZ has a substantial and increasing global disease burden accounting for 1.5% of disability-adjusted life years in the 25–49-year age group (Vos et al., 2020). Although the pathophysiology of the disease is still not understood, recent genomic studies suggest an association with genes expressed in excitatory and inhibitory neurons in the brain (Trubetskoy et al., 2022). Fine mapping of the associated genes such as the NMDA receptor subunit GRIN2A showed a concentration of the associated genes in pre- and postsynaptic locations (Trubetskoy et al., 2022). These data are in accordance with the postulated interneuron hypothesis of schizophrenia (Lewis et al., 2005) supposing a primary disturbance of the rhythm-generating fast-spiking parvalbumin positive perisomatic inhibitory interneurons (PV INs) in the cortex. The subsequent abnormal local field potential rhythms at gamma and beta frequencies are suggested to be the central pathophysiological trait of schizophrenia, responsible for the negative, positive and cognitive symptoms of the disease (Lee et al., 2003; Lewis et al., 2005). Indeed, increased resting-state and task induced gamma oscillations have been found in patients with schizophrenia (Teale et al., 2008; Donkers et al., 2013; Hirano et al., 2015) and different disease models in animals (Jadi et al., 2016; Speers and Bilkey, 2021) such as acute application of the NMDA receptor antagonist MK-801 (Kehrer et al., 2007; Lemercier et al., 2017). Modulating these oscillatory microcircuits in combination with alternative approaches such as cognitive training and brain stimulation was suggested to represent novel and selective interventions for improving cognition in schizophrenia (Sohal, 2022).

Depressive disorders possess the sixth highest disease burden globally responsible for 3.5% of disability-adjusted life years in the 25–49-year age group (Vos et al., 2020). Although significant advances have been achieved understanding its pathophysiology, no universally accepted mechanism can explain all symptoms of the disease (Otte et al., 2016). Serotonin reuptake inhibitors, the pharmacological backbone of the treatment, are only effective in one-thirds of patients and show therapeutic effects after couple of weeks (Rush et al., 2006). Recently, the focus was shifted to therapeutics with shorter latency such as the NMDA receptor antagonist ketamine. Indeed, while initial models focused on neurochemical changes in the brain, like the monoamine hypothesis, more recent research turned toward a network mechanism to explain the key symptoms of the disease (Spellman and Liston, 2020). Preclinical and clinical data show an overstimulation of NMDA receptors within these dysfunctional neuronal networks (Adell, 2020; Johnston et al., 2024) and that antidepressants have inhibitory effects on NMDA receptors (Szasz et al., 2007). Conforming to this, several studies reported that cortical GABAergic inhibitory circuits and gamma oscillations are impaired in patients with depression or in animal models of the disease and gamma oscillations were suggested as a biomarker for major depression (Mendez et al., 2012; Fitzgerald and Watson, 2018; Liu et al., 2023).

The opposite use of the same substance family in schizophrenia and depression raises the question how the blockade of NMDA receptors changes important biomarkers of these two psychiatric diseases and if these changes are in accordance with the observed disease pathophysiology. Previous studies have investigated the effect of acutely applied NMDA receptor antagonists on cortical gamma oscillations (Kehrer et al., 2007; Lemercier et al., 2017). However, it remained unclear how a long-term administration scheme, as practiced in depressive patients, affects cortical gamma oscillations. Since women are twice as likely to be diagnosed with depression compared to men (Keers and Aitchison, 2010) it is also important to examine whether differences between females and males exist regarding the effects of NMDA receptor antagonists. To shed light onto these questions, the present study was planned to investigate the sex specific effects of chronic application of the NMDA receptor antagonist MK-801 on hippocampal gamma oscillations and its behavioral counterparts. Our results show that subchronic NMDA receptor antagonist administration increased the power of hippocampal gamma oscillations predominantly in females and this change was accompanied with decreased novel object recognition memory and increased stereotypy suggesting that female rats are more sensitive to the effects of the NMDA receptor antagonist MK-801.

## 2 Materials and methods

### 2.1 Animals

**Figure 1:**
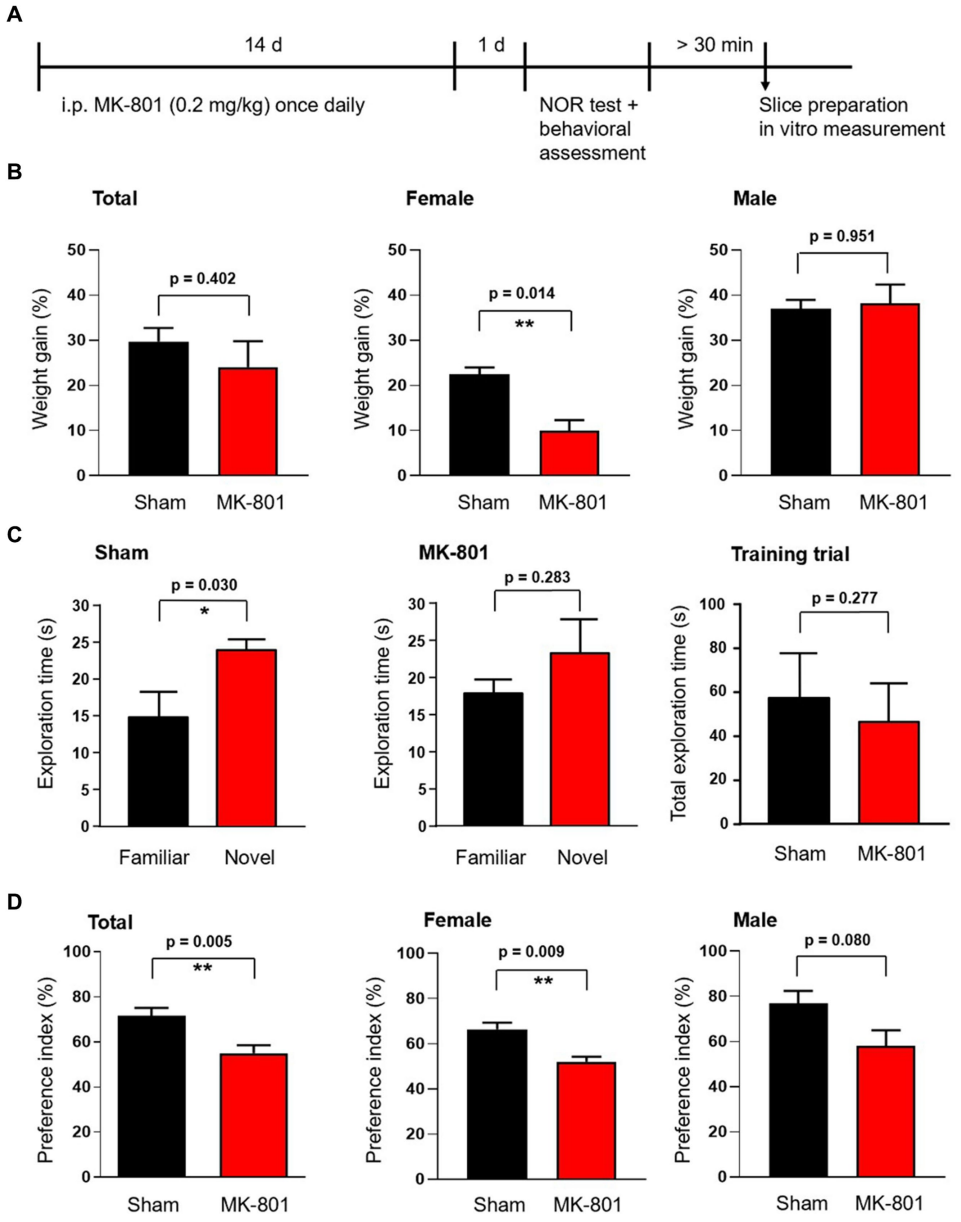
Effect of subchronic MK-801 treatment on the novel object recognition memory of rats. **(A)** Overview of the treatment scheme and time points of behavioral and *ex vivo* tests. **(B)** Bar graphs show the weight gain of animals during the 14-days long application of MK-801 (red) or saline (sham, black). Weight gain was significantly reduced in female animals but not in males. **(C)** Bar graphs show the exploration times spent exploring the familiar and novel objects in the novel object recognition (NOR) test. Sham treated rats explored novel objects significantly longer than the already known familiar object (left). MK-801 treatment diminished the difference in exploration time (middle). Sham and MK-801 treated animals explored both (identical) objects in the first training trial similarly (right). **(D)** MK-801 significantly reduced the exploration time spent with the novel object, expressed as novelty preference index (time spent by exploring of the novel object divided by the total exploration time of both objects expressed as percentage, left). The reduction of the preference index by MK-801 was significant in female rats and not in males. Data are mean ± S.E.M, *p < 0.05, **p < 0.01.

All procedures were conducted in accordance with the guidelines of the European Communities Council and the institutional guidelines approved by the Berlin Animal Ethics Committee (Landesamt für Gesundheit und Soziales Berlin, G0036/17). Wistar rats were kept in groups of two individuals of same sex and experimental group (treatment or sham group) in one cage. Animals were housed on a 12/12 h light/dark cycle with *ad libitum* food and water. Both sexes of animals from an age of P60 were injected daily for 14 days with an aqueous solution of MK-801 maleate (0.2 mg/kg, 4 male and 4 female rats) in the treatment group or with a sterile 0.9% NaCl solution in the sham group (4 male and 4 female rats). The last i.p. injections were done 24 h before starting the experiments (Figure 1A). While the effects of acute administration of MK-801 on hippocampal gamma oscillations were intensively investigated both *in vitro* (Kehrer et al., 2007; Lemercier et al., 2017) and *in vivo* (e.g., Sullivan et al., 2015), the hippocampal effects of a longer chronic or subchronic application was previously not tested *in vitro*.

### 2.2 Novel object recognition (NOR) test

The NOR test (Ennaceur, 2010) was carried out according to the protocol by Mathiasen and DiCamillo (2010). Twenty-four hours after the last injection, rats were individually transferred into a separate room and left to acclimatize for 15 min in a resting cage. Before starting the tests, a rat that was not part of the experiment was placed into the test box for 15 min to saturate the box and all objects within with its odor signal. The goal of this was to eliminate exogenous olfactory stimuli in the test box and between objects that would influence the exploration of the objects and their odor-related identification. The experiment was split into two test trials. In the first training trial, two identical objects in standard distance were placed in the test box. The rat was put into the test box for 3 min and recorded on video for the whole time. After 3 min, the animal was removed and put back into the resting cage. One of the objects was exchanged for a novel object. The second test trial started after an intertrial interval of 15 min. The rat was placed for 3 min into the test box and again recorded on video for 3 min. The novel object and side of the novel object within the cage was randomly defined. Animals with no cognitive impairment explore the novel object longer compared to the familiar object. Two independent persons blinded measured the absolute times the rats spent at each object by using the recorded videos. Two parameters were calculated: the exploration time at the objects and the novelty preference index (the time spent by exploring of the novel object divided by the total exploration time of both objects expressed as percentage). Data are presented as mean ± S.E.M. Statistical comparisons of the parameters between sham and MK-801-treated animals were made using Welch t-test. Significance was set at *p* = 0.05.

### 2.3 Behavioral assessment

For the determination of psychomotor activity, we used the recorded videos of the NOR testing and analyzed them by measuring the duration of time rats spent with three different types of activity during the first trial. First, locomotor activity was defined as the fraction of time (%) during the 3-min test period spent in locomotion. Second, stereotypy was defined as time fraction (%) spent on head-weaving (>2 times side-to-side motion of the head reaching more than 30 degrees from the body axis) or circling (closed loop of locomotion) or axial turning (circling movements of the forepaws with the haunches remaining still). Third, grooming was defined as the fractional time spent with licking or preening any part of the body (Feinstein and Kritzer, 2013). The sample size was the number of animals. Group size was estimated by the program G*Power3 (Faul et al., 2007). Data are presented as mean ± S.E.M. Statistical comparisons of the parameters between sham and MK-801-treated animals were made using Student’s t-test. Significance was set at *p* = 0.05.

### 2.4 Materials

MK-801, Physostigmine (Physo) and kainate (KA) were ordered from Tocris Bioscience (Bristol, UK). Acetylcholine (ACh) was delivered from Sigma-Aldrich (Taufkirchen, Germany).

### 2.5 Slice preparation

After the behavioral testing, the rats were transported from the animal house to the preparation room and left for at least 30 min to acclimatize. Afterwards, they were decapitated under isoflurane anesthesia. Their brain was immediately removed and placed in an ice-cold sucrose-based and carbogenated (95% O_2_ / 5% CO_2_) solution with the following composition (in mM): sucrose, 85; NaCl, 80; NaHCO_3_, 25; NaH_2_PO_4_, 1.25; KCl, 2.5; glucose, 25; CaCl_2_, 0.5; MgCl_2_, 3. The hemispheres were cut into 400-μm thick horizontal slices containing the formation of the hippocampus by a vibratome (Leica VT 1200S, Leica Mikrosysteme Vertrieb GmbH, Wetzlar, Germany). The slices were immediately transferred to an interface-type recording chamber and constantly perfused with warm (35°C) and carbogenated artificial cerebrospinal fluid (ACSF) with a flow rate of 1.7 mL/min and a composition (mM) of NaCl, 129; KCl, 3; NaHCO_3_, 21; NaH_2_PO_4_, 1.25; MgSO_4_, 1.8; CaCl_2_, 1.6; glucose, 10. The slices were incubated for at least 30 min in the recording chamber before starting the experiment.

### 2.6 Recording of gamma oscillations

Gamma oscillations were induced by bath application of either KA (150 nM) or Ach and Physo (10 μM and 2 μM, respectively; Schulz et al., 2012a). Local field potentials (LFPs) were recorded by silver chloride electrodes in micropipettes (ACSF filled, resistance ~3 MΩ) placed in the stratum pyramidale of the hippocampal CA3 area as described previously (Wildner et al., 2024). Recordings were amplified by an EXT-2 (NPI electronic, Tamm, Germany) amplifier, analog low pass filtered at 1 kHz and sampled at 5 kHz by a CED 1401 interface (Cambridge Electronic Design, Milton, UK). For the analysis of pharmacologically induced hippocampal gamma oscillations, power spectra were calculated every 2 min with a 120-s window throughout the recording (Lemercier et al., 2016). From these, the power spectra, peak power, peak frequency, and the quality factor (Q-factor) of the oscillations were determined later by the usage of the Spike2 software. The Q-factor of the gamma oscillations was calculated by the equation: Q = f/B, where f is the peak frequency and B is the bandwidth at 50% of maximum peak power (Meier et al., 2020). Neuronal activity in the power spectrum was considered an oscillation if the Q factor was subcritical (above 0.5) (Lemercier et al., 2017). For the comparison between treated and non-treated groups, the above parameters between 90 and 100 min after gamma oscillation stimulation were compared between slices from MK-801 and sham-treated animals. For the analysis of hippocampal oscillations, a second-order Butterworth band-stop (49.5–50.5 Hz) filter was used to remove 50 Hz noise. Since the power of gamma oscillations is lognormally distributed, peak power (μV^2^) is shown as geometric mean with the geometric standard deviation factor (GeoSD) (Klemz et al., 2021) calculated by GraphPad Prism (Boston, MA, USA). Sample size n was defined as individual slices. Group size estimation was done by the program G*Power3 (Faul et al., 2007). The peak frequency (Hz) is represented as mean ± SEM. For the statistical comparisons of the parameters, we used the t-test. The lognormally distributed power values were first log transformed and then statistically analyzed by Student’s t-test (Klemz et al., 2022). Significance level was set at *p* = 0.05.

## 3 Results

### 3.1 Effects of subchronic MK-801 application on the behavior

To assess the action of MK-801 in female and male rats, we first measured the weight changes of the animals during the subchronic (14 days) application of MK-801 at subanesthetic doses (0.2 mg/kg). During the 2-weeks treatment, MK-801 significantly decreased the relative weight gain of female animals compared to sham-treated controls (10.0 ± 2.3% versus 22.4 ± 1.6%, respectively, *p* = 0.006; *n* = 4 in both groups; Figure 1B). We did not observe the same effect in male animals (MK-801: 38.1 ± 4.2%, *n* = 4; sham: 37.0 ± 1.9%, *p* = 0.823; *n* = 4; Figure 1B).

The novel object recognition (NOR) test is widely used to model recognition memory (Ennaceur, 2010). Sham (saline) treated animals spent in the test trial significantly more time exploring the novel object compared to the familiar object (24.0 ± 1.4 s and 14.7 ± 3.6 s, respectively, *n* = 8, *p* = 0.030; Figure 1C). MK-801-treated animals failed to significantly differentiate between the novel and familiar object and spent comparable time at each (23.3 ± 4.5 s and 17.8 ± 2.0 s, respectively, *n* = 8, *p* = 0.283; Figure 1C). MK-801 might have decreased the novelty preference of the animals because it lowered the object exploration duration in the training trial and herewith the encoding time. To test this possibility we calculated the total exploration times in the training trial in sham and MK-801-treated animals and did not find statistical difference (57.2 ± 7.3 s and 46.4 ± 6.2 s, respectively, *n* = 8, *p* = 0.277, Figure 1C right). Comparing the novelty preference indices (the time spent exploring the novel object divided by the total exploration time in the test trial) revealed that MK-801-treated rats spent significantly less time with exploring the novel object compared to the sham-treated animals (*p* = 0.005; Figure 1D). The sex specific analysis revealed a significant effect of MK-801 only in female rats (66.4 ± 2.9% and 52.0 ± 2.3% in MK-801 and sham, respectively, *n* = 4 in both groups, *p* = 0.009; Figure 1D), while male rats showed only a tendency (76.9 ± 5.5% and 57.9 ± 7.0%, respectively, *n* = 4 in both groups, *p* = 0.080; Figure 1D).

**Figure 2:**
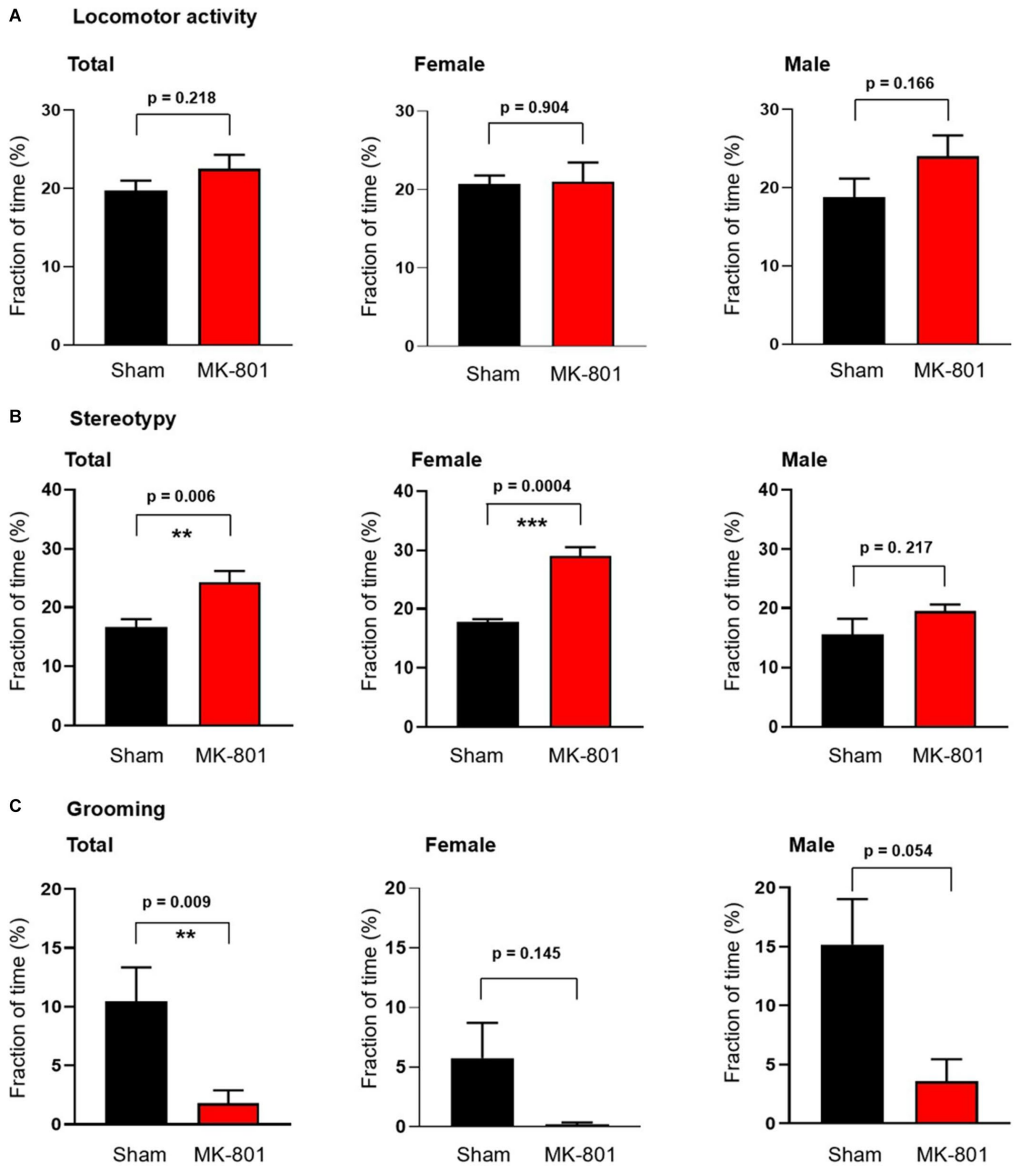
Sex-specific behavioral effects of subchronic MK-801 treatment. **(A)** MK-801 had no effect on the locomotor activity. **(B)** 14-day long treatment with MK-801 increased stereotypy in female animals but not in males. **(C)** MK-801 decreased grooming behavior. Data are shown as mean ± S.E.M, **p < 0.01, ***p < 0.001.

We investigated next the effect of MK-801 on the behavior of the rats and found that MK-801 treatment did not change the locomotor activity of both female and male animals (Figure 2A). In contrast, we observed an increase of the duration of stereotypic behavior from 16.7 ± 1.3% to 24.3 ± 2.0% (*p* = 0.006; *n* = 8 in both groups, Figure 2B). This increase was only observed in female rats (from 17.8 ± 0.5% to 29.0 ± 1.5%, *p* = 0.0004) and not in males (15.5 ± 2.7% vs. 19.5 ± 1.1%, *p* = 0.217; Figure 2B). Measuring the time rats spent with grooming revealed a significant decrease from 10.5 ± 2.9% to 1.9 ± 1.1% (*p* = 0.009, *n* = 8) without an observed sex difference (Figure 2C).

In summary, subchronic low and subanesthetic application of MK-801 had a significant behavioral effect characterized by a reduced weight gain during the 14 days of treatment, an impaired novel object recognition, increased stereotypic behavior and decreased grooming, predominantly in female animals.

### 3.2 Effects of subchronic MK-801 on *in vitro* hippocampal gamma oscillations

**Figure 3:**
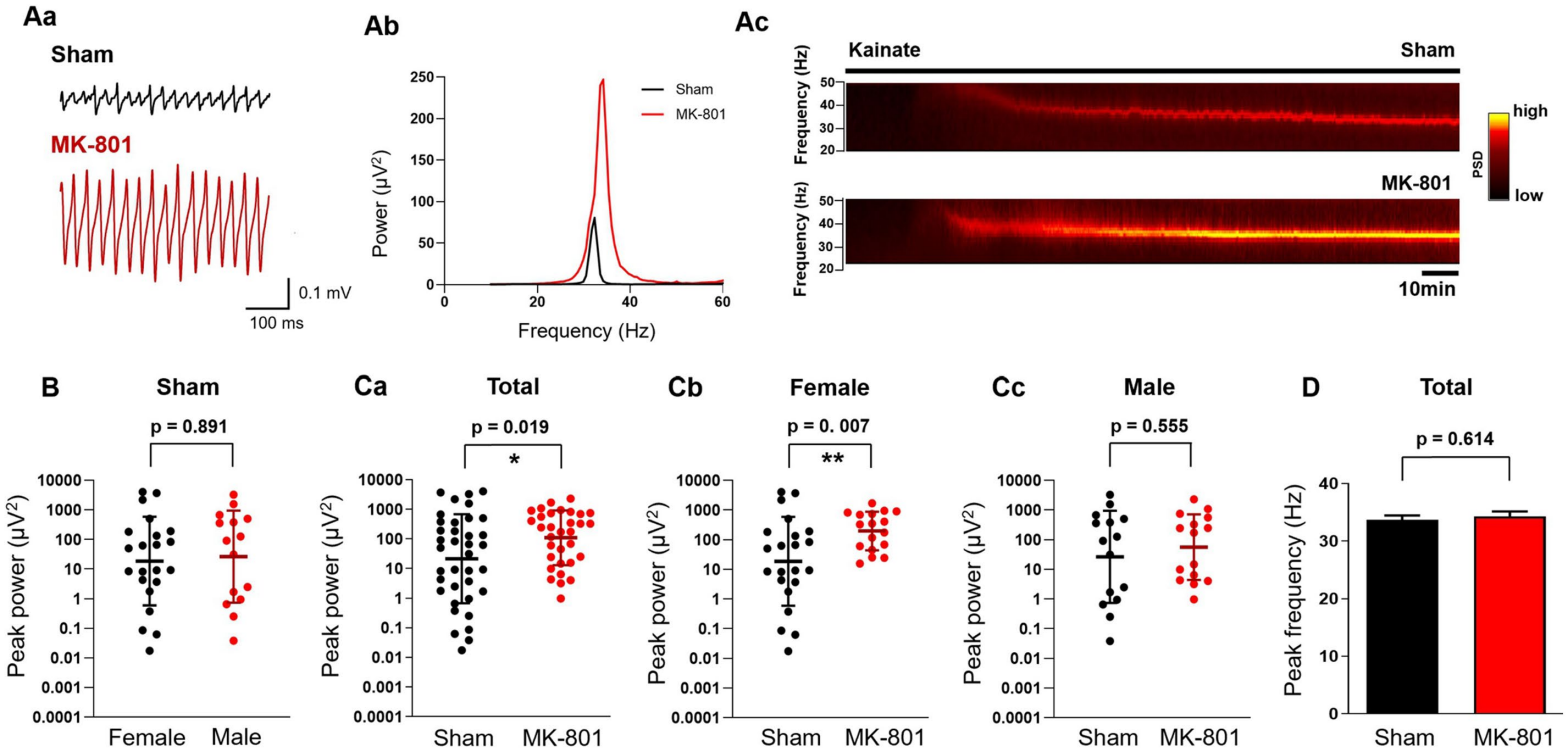
Subchronic MK-801 treatment increased the power of *ex vivo* gamma oscillations induced by bath application of kainate (KA). **(Aa)** Representative local field potential traces in hippocampal CA3 slices of sham and MK-801 treated rats after the application of KA (150 nM). **(Ab)** Representative power spectra of KA-induced hippocampal gamma oscillations from sham (black) and MK-801 (red) treated animals. **(Ac)** Representative spectrograms from sham (black) and MK-801 (red) treated animals. Horizontal bar indicates the duration of KA application. **(B)** Scatter plots of the peak power of KA-induced gamma oscillations in female (black) and male (red) sham treated animals. Bars indicate geometric mean and geometric standard deviation factor. **(Ca)**: MK-801 (red) increased the peak gamma power compared to sham (black) treated animals. Bars indicate geometric mean and geometric standard deviation factor. MK-801 (red) increased the peak power of gamma oscillations compared to sham (black) treatment in female rats **(Cb)** but not in males **(Cc)**. Bars indicate geometric mean and geometric standard deviation factor. **(D)** MK-801 did not affect the peak frequency of gamma oscillations. PSD, Power spectral density; *p < 0.05, **p < 0.01.

Local *in vitro* gamma oscillations can be induced pharmacologically by activation of KA and muscarinic receptors (Fisahn et al., 1998, 2004). Because these gamma oscillations are probably generated by different networks (Bartos et al., 2007) and can be modulated differentially (Schulz et al., 2012b) we tested the effects of MK-801 administration on both variants. The peak power of KA-induced gamma oscillations in hippocampal slices from female and male sham-treated rats did not differ (geometric mean, geometric SD factor: 18.7 μV^2^, 31.4 μV^2^, *n* = 22 and 26.5 μV^2^, 35.8 μV^2^, *n* = 15, respectively, *p* = 0.891; Figure 3B). MK-801 treatment significantly increased the peak power (geometric mean, geometric SD factor: 21.5 μV^2^, 31.7 μV^2^, *n* = 37 and 109.8 μV^2^, 8.3 μV^2^, *n* = 32, respectively, *p* = 0.019; Figures 3A,Ca). When analyzed for sex-specific effect, we observed an increase of power only in female rats (sham: geometric mean, geometric SD factor: 18.7 μV^2^, 31.4 μV^2^, *n* = 17; MK-801: 197.7 μV^2^, 4.5 μV^2^, *n* = 22; *p* = 0.007; Figure 3Cb) while in male animals MK-801 did not have an effect (sham: geometric mean, geometric SD factor: 26.54 μV^2^, 35.84 μV^2^, *n* = 15; MK-801: 56.39 μV^2^, 12.82 μV^2^, *n* = 15, respectively, *p* = 0.555; Figure 3Cc). We observed neither difference or sex-specific effects in the peak frequency of the induced gamma oscillations from sham and MK-801 treated animals (33.7 ± 0.8 Hz and 34.3 ± 0.9 Hz, respectively, *p* = 0.614; Figure 3D).

**Figure 4:**
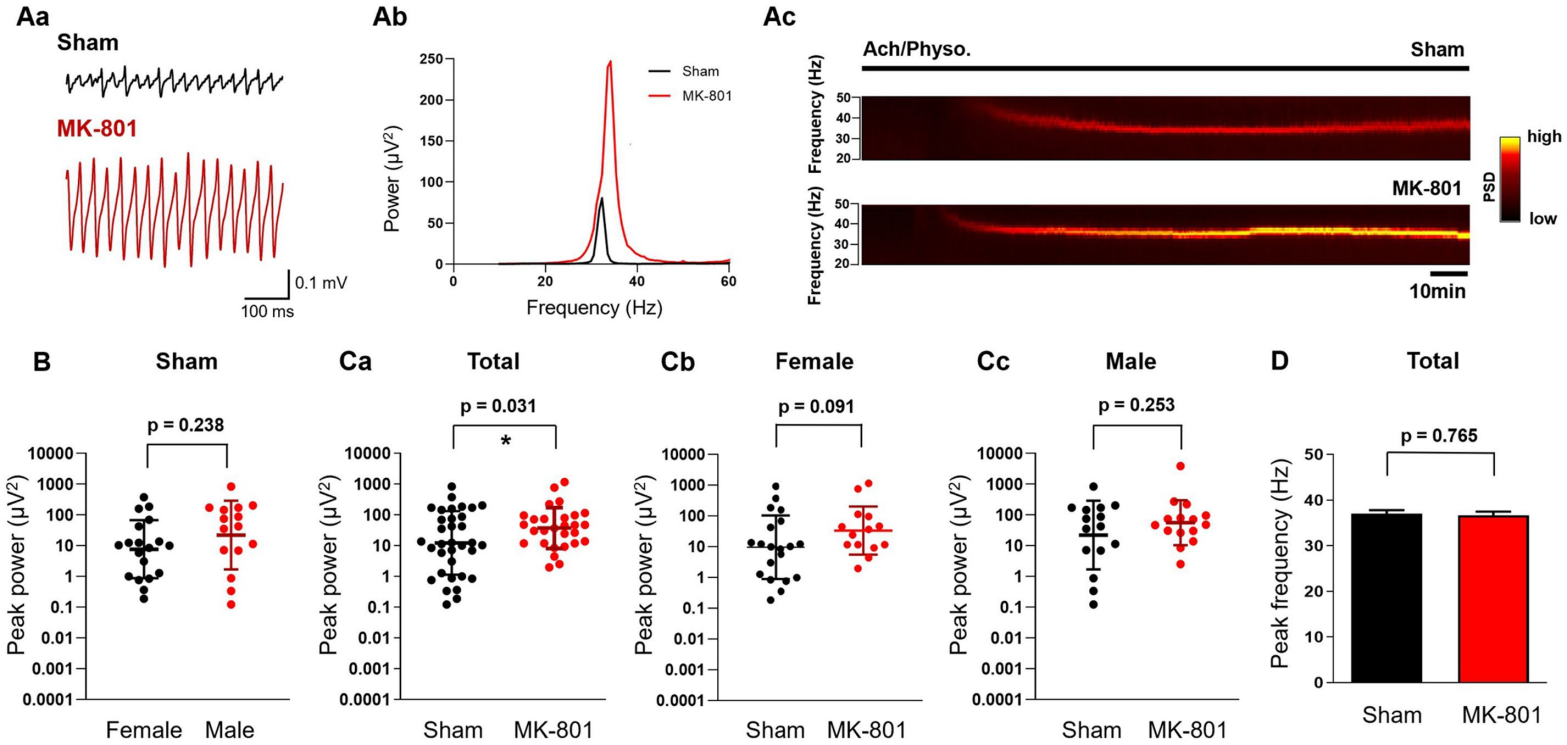
Subchronic MK-801 treatment increased the power of *ex vivo* gamma oscillations induced by bath application of ACh and Physo. **(Aa)** Representative local field potential traces in hippocampal CA3 slices of sham and MK-801 treated rats after the application of ACh (10 μM) & Physo (2 μM). **(Ab)** Representative power spectra of cholinergically induced hippocampal gamma oscillations from sham (black) and MK-801 (red) treated animals. **(Ac)** Representative spectrograms from sham (black) and MK-801 (red) treated animals. Horizontal bar indicates the duration of Ach/Physo application. **(B)** Scatter plots of the peak power of cholinergically induced gamma oscillations in female (black) and male (red) sham treated animals. Bars indicate geometric mean and geometric standard deviation factor. **(Ca)** MK-801 (red) increased the peak gamma power compared to sham (black) treated animals. Bars indicate geometric mean and geometric standard deviation factor. **(Cb)** Peak gamma oscillation power in sham (black) and MK-801 (red) treated female rats. Bars indicate geometric mean and geometric standard deviation factor. **(Cc)** Peak power of gamma oscillations in sham (black) and MK-801 (red) treated male rats. Bars indicate geometric mean and geometric standard deviation factor. **(D)** MK-801 did not affect the peak frequency of gamma oscillations. PSD, Power spectral density; *p < 0.05.

Next, we induced gamma oscillations in hippocampal slices with ACh (10 μM) and Physo (2 μM). The peak power values did not differ in sham treated female and male rats (geometric mean, geometric SD factor: 7.7 μV^2^, 8.8 μV^2^, *n* = 19 and 22.2 μV^2^, 13.0 μV^2^, *n* = 15, respectively, *p* = 0.238; Figure 4B), similar to findings in mice (Guneykaya et al., 2023). Subchronic treatment with MK-801 increased the power of cholinergically induced gamma oscillations (geometric mean, geometric SD factor: 44.0 μV^2^, 5.6 μV^2^, *n* = 29) compared to sham treatment (13.9 μV^2^, 11.6 μV^2^, *n* = 35; *p* = 0.031; Figures 4A,Ca). The effect of MK-801 was stronger in female animals compared to males, although it did not reach the significance level (sham: geometric mean, geometric SD factor: 9.8 μV^2^, 10.7 μV^2^, *n* = 20; MK-801: 34.1 μV^2^, 6.1 μV^2^, *n* = 14; *p* = 0.091; Figure 4Cb). In male rats, MK-801 did not increase the power (sham: geometric mean, geometric SD factor: 22.2 μV^2^, 13.0 μV^2^, *n* = 15; MK-801: 56.0 μV^2^, 5.3 μV^2^, *n* = 15, respectively, *p* = 0.253; Figure 4Cc). MK-801 treatment did not affect the peak frequency of the oscillations (sham: 37.0 ± 0.8 Hz, *n* = 35 and MK-801 36.7 ± 0.8 Hz, *n* = 29, respectively, *p* = 0.765; Figure 4D) and we did not observe a sex-specific effect of MK-801 on the peak frequency (not shown).

Thus, subchronic MK-801 treatment increased the power of both KA- and acetylcholine induced gamma oscillations in the hippocampus. KA-induced gamma oscillations were clearly sex-specific and significantly increased only in females rats. Cholinergic gamma oscillations showed only a tendency for sex-specificity.

## 4 Discussion

The aim of this study was to evaluate the effects of subchronic application of the NMDA antagonist MK-801 on the behavior and hippocampal local network activity in rats. We found that a 14-day subanesthetic treatment with MK-801 enhanced the amplitude of hippocampal gamma oscillations. This effect was seen predominantly in female animals and was associated with increased stereotypy, decreased grooming and impaired recognition memory.

Gamma oscillations reflect rhythmic neuronal network activity that is synchronized across neurons and brain areas (Buzsáki and Wang, 2012). They can be registered by scalp electroencephalography (EEG), electrocorticography, magnetoencephalography or by measuring local field potentials (LFPs) intracranially in the brain tissue. In addition, since gamma oscillations are generated locally by an interplay between pyramidal cells and parvalbumin positive interneurons (Hájos and Paulsen, 2009) it is possible to induce and measure them *ex vivo* in acutely isolated brain slices by means of LFP measurements (Fisahn et al., 1998; Schulz et al., 2012b). Pharmacologically induced *ex vivo* gamma oscillations in the CA3 were shown to correlate with *in vivo* neuronal activity and space reference memory (Lu et al., 2011) confirming that they are a useful measure of *in vivo* network activity and associated behavior in brain slices offering the advantage of investigating real local processes.

Gamma oscillations are proposed to coordinate fundamental neuronal processes underlying cognition (Fries, 2009) and have been associated with higher cognitive functions such as perception, attention, learning and memory (Bosman et al., 2014). In the hippocampus, they play a role in working memory (Yamamoto et al., 2014), in memory replay (Carr et al., 2012) as well as in novel object recognition (Trimper et al., 2014, 2017). The novel object recognition test is widely used to model recognition memory (Ennaceur, 2010). The hippocampus operates as a novelty detector comparing incoming and stored information which might be a key function of exploratory behavior during discovering the environment (Honey et al., 1998; Johnson et al., 2012). The hippocampus is involved in the novel object recognition memory when a delay longer than 10 min is entailed between the first and the second trials of NOR (Cohen and Stackman, 2015). Our results, using 15 min delay between the trials, showed that MK-801 affected both hippocampal gamma oscillations and NOR, including the predominant effect in female animals. This suggests that the increased power of hippocampal gamma oscillations is associated with the impaired recognition memory.

While gamma oscillations play a key role in cognition, their malfunction was observed both in schizophrenia and depressive disorders. In schizophrenia patients, baseline resting-state and task-induced gamma oscillations are increased similar to gamma oscillations in animal models (Teale et al., 2008; Donkers et al., 2013; Hirano et al., 2015; Lemercier et al., 2017; Hasam-Henderson et al., 2018). On the contrary, steady-state gamma responses evoked by repetitive sensory, in most cases auditory stimuli are decreased in both patients and animals studies (Sivarao et al., 2013; Thuné et al., 2016; Choi et al., 2023). While the power of induced gamma oscillations correlated with cognitive performance (Johannesen et al., 2016; Perrottelli et al., 2022) most studies reported no significant associations between the steady state gamma response and cognition in schizophrenia patients (Light et al., 2006; Kirihara et al., 2012; Perrottelli et al., 2022). An explanation for the opposing change of gamma oscillation power could be that pathologically increased baseline gamma power preceding the trial might limit the ability of bottom-up sensory transmission evoked gamma activity to further increase. This might impede the encoding of new information leading to reduced trial accuracy (Johannesen et al., 2016). We modeled the disease with a 14-day long i.p. application of 0.2 mg/kg MK-801 to adult rats. In contrast to single dose NMDA antagonist applications modeling acute first episode schizophrenia (Kehrer et al., 2007; Sullivan et al., 2015; Lemercier et al., 2017) the longer application is supposed to simulate a chronic disease state in animals (McNally et al., 2013). To test the effect of MK-801 on the behavior we analyzed the locomotor activity, stereotypy and grooming of the of the animals. While hyperlocomotion and stereotypy are considered as surrogates for the positive symptoms of schizophrenia, reduced grooming models the negative signs (Feinstein and Kritzer, 2013). In our experimental setup, subchronic treatment with MK-801 increased stereotypy only on female rats, in line with previous studies showing more pronounced effects in this sex (Hönack and Löscher, 1993; Andiné et al., 1999; Devaud, 2003). Subchronic MK-801 treatment did not significantly suppressed grooming in females which is in agreement with earlier findings after acute application (Feinstein and Kritzer, 2013). Although a profound hyperlocomotor activity was observed after acute application of MK-801 in females (Feinstein and Kritzer, 2013), we did not observe a treatment effect after subchronic MK-801 application presumably because the induction of stereotyped behavior counteracted ambulatory activity (van den Buuse, 2010). The results suggest that subchronic application of MK-801 daily induced a behavioral phenotype in the treated rats resembling both positive and negative symptoms of schizophrenia.

Present study investigated for the first time the effect of subchronic MK-801 on *ex vivo* pharmacologically induced hippocampal gamma oscillations. While the effects of acute systemic application of MK-801 on hippocampal gamma oscillations was intensively investigated in animal studies both *in vitro* (Kehrer et al., 2007; Lemercier et al., 2017) and *in vivo* (e.g., Sullivan et al., 2015; Cui et al., 2022), a longer application was previously not tested *in vitro* in the hippocampus. In prefrontal cortex slices, five-day-long MK-801 application decreased the power of gamma oscillations (McNally et al., 2013). *In vivo* measurements from the hippocampus of rats showed no effects of a 21-day-long MK-801 treatment on the hippocampal gamma (Sullivan et al., 2015). We found that MK-801 increased the gamma power in hippocampal slices indicating that *ex vivo* induced gamma oscillations response to systemic MK-801 application similar to baseline resting-state and task-induced gamma oscillations measured in schizophrenia patients. Our results suggest that modeling schizophrenia with systemic application of an NMDA receptor antagonist and the subsequent registration of pharmacologically induced *ex vivo* gamma oscillations in the hippocampus reproduces electrophysiological traits found in patients and can used as a biomarker for further research and pharmacological testing.

NMDA receptor antagonists, on the other hand, are used in the treatment of depressive disorders. Recent studies described reduced resting-state pre-task gamma oscillations in patients (Pizzagalli et al., 2006; Wang et al., 2023), and emotional task induced and steady-state evoked gamma oscillations were also found to be impaired (Lee et al., 2010; Liu et al., 2014, 2023). Animal models of depression induced with chronic unpredictable mild stress showed decreased auditory steady-state gamma responses (He et al., 2023). In last years, gamma oscillations have been increasingly suggested as a novel biomarker for major depression (Fitzgerald and Watson, 2018; He et al., 2023; Liu et al., 2023). Our results indicate that a longer application of the NMDA receptor antagonist MK-801 significantly increases the power of gamma oscillations suggesting that pharmacological treatment with an NMDA antagonist might normalize the reduction of the power observed in both patients and animal models. These findings are in line with experiments showing that memantine, an NMDA receptor antagonist used in the treatment of Alzheimer’s disease, also increased the power of hippocampal and neocortical gamma oscillations (Guadagna et al., 2012; Ahnaou et al., 2014; Ma et al., 2015).

The mechanism behind the increased gamma power after NMDA receptor antagonism currently remains unknown, however, two main hypotheses are considered to play a role: the theory of direct inhibition of cortical pyramidal cells and the disinhibition theory (Miller et al., 2016). The direct inhibition hypothesis postulates that an antagonism of extrasynaptic NMDARs on pyramidal cells, normally activated by low-level ambient glutamate, induces activity-independent homeostatic plasticity leading to increased excitatory synaptic inputs onto these neurons and increased gamma oscillations (Miller et al., 2016; Petzi et al., 2023). The disinhibition theory suggests that antagonism or dysfunction of the NMDA receptors on INs decreases the inhibitory tone in the circuit resulting in a higher neuronal activity within the network (Molina et al., 2014; Miller et al., 2016; Johnston et al., 2024). This theory assumes that NMDA receptors are either predominantly expressed on cortical INs compared to pyramidal cells (Lisman et al., 2008; Povysheva and Johnson, 2016) or are functionally more conductive, because the most abundant IN type in the cortex, the PV INs, are more depolarized and their receptors are thus less blocked by Mg^2+^ (Grunze et al., 1996; Homayoun and Moghaddam, 2007). This would mean that antagonists might influence this cell type more effectively. Because of their reduced excitability by NMDA receptor antagonism, PV INs require more synchronized inputs resulting in more synchronous perisomatic inhibition and enhanced gamma oscillation power (Spencer, 2009; Jadi et al., 2016).

An important finding of our study is the sex specificity of the MK-801 effect on hippocampal gamma oscillations. Previous studies tested only male animals (Sullivan et al., 2015; Cui et al., 2022) or did not investigate sex effects (Kehrer et al., 2007; Lemercier et al., 2017). Our study is the first that investigated the effect of NMDA receptor antagonists in female and male animals on hippocampal gamma oscillations and shows that MK-801 had a significantly stronger network effect in female rats compared to males. These observations are in accordance with the sex-specific behavioral effects observed in this and previous studies (Hönack and Löscher, 1993; Andiné et al., 1999; Devaud, 2003; Feinstein and Kritzer, 2013). We have two possible explanations for the sex difference. First, the ovarian hormone estrogen in female animals inhibits the expression of cytochrome P450 2D6 (CYP2D6) (Wang et al., 2014; Konstandi et al., 2020), which is the predicted primary metabolizing enzyme of MK-801 (Banerjee et al., 2020). It can be proposed that the applied MK-801 reached higher levels in female animals. Second, estrogen increases the expression of NMDA receptors in the membrane of neurons (Weiland, 1992; El-Bakri et al., 2004; Ikeda et al., 2010) and enhances the NMDA receptor dependent synaptic currents and calcium transients on hippocampal neurons (Foy et al., 1999; Pozzo-Miller et al., 1999). Increased expression of NMDA receptors in the membranes might cause more intense effects of NMDA receptor antagonists as seen in our experiments. A recent study has shown that besides pyramidal cells also PV INs express aromatase (Hernández-Vivanco et al., 2022), the enzyme to produce estradiol (Do Rego et al., 2009). Under these circumstances, NMDA receptor antagonists might have a stronger direct inhibitory or disinhibition effect on the network as discussed above. These considerations might also explain why KA-induced gamma oscillations had a stronger sex-specificity. Since KA receptors seem to preferentially activate interneurons compared to muscarinic ACh receptors (believed to be predominantly expressed on principal cells), KA-induced gamma oscillations are postulated to be generated by a primary stimulation of interneurons (Bartos et al., 2007). The disinhibition theory postulates that interneurons express more functional NMDA receptors, which might be further increased by estrogen in female animals. Thus, NMDA receptor antagonists may disinhibit the interneuron-based KA-induced gamma oscillations in female animals more effectively. Preclinical studies have shown that female animals are more sensitive to treatment with ketamine compared to males and develop more severe side effects (Ponton et al., 2021). These results are not consistent with the existing few human data, where the treatment response after systemic acute low dose ketamine application seems to be similar in women and men (Coyle and Laws, 2015; Freeman et al., 2019; Ponton et al., 2021). The sex effect of ketamine, however, might depend on the dose applicated because higher (1 mg/kg) dose reduced the Hamilton Depression Rating Scale scores more in women than in men (Freeman et al., 2019). In contrast to the treatment response, the side effects show clear sex-specific differences (Liebe et al., 2017; Ponton et al., 2021) indicating that ketamine acts sex-specifically also in human. Our results suggest that the higher sensitivity in females is not specific to ketamine but seems to be a generic characteristic trait of NMDA receptor antagonists.

In conclusion, presented data demonstrate that longer systemic application of the highly selective, non-competitive, use-dependent NMDA receptor antagonist MK-801 increases the amplitude of hippocampal gamma oscillations. The effects are associated with behavioral alterations such as impaired recognition memory, increased stereotypy and decreased grooming, surrogate symptoms of the cognitive, positive and negative symptoms of schizophrenia, respectively. Both the electrophysiological and behavioral changes were predominantly observed in female animals suggesting a sex-specific effect of NMDA receptor antagonists. The results reproduce electrophysiological and behavioral traits found in patients and in other schizophrenia animal models and suggest that hippocampal gamma oscillations can be used as a biomarker for further research and pharmacological testing.

## Data availability statement

The raw data supporting the conclusions of this article will be made available by the authors, without undue reservation.

## Ethics statement

The animal study was approved by Landesamt für Gesundheit und Soziales, Berlin, Germany. The study was conducted in accordance with the local legislation and institutional requirements.

## Author contributions

TN: Formal analysis, Investigation, Project administration, Visualization, Writing – original draft, Writing – review & editing. ZG: Conceptualization, Formal analysis, Methodology, Resources, Supervision, Visualization, Writing – original draft, Writing – review & editing.
